# Student health behavior and academic performance

**DOI:** 10.7717/peerj.11107

**Published:** 2021-04-16

**Authors:** Peter R. Reuter, Bridget L. Forster

**Affiliations:** Marieb College of Health & Human Services, Florida Gulf Coast University, Fort Myers, Florida, United States of America

**Keywords:** Student health behavior, College students, Grade point average, Academic performance, Healthy eating, Sleep, Working, Vaping, Breakfast, Fast food

## Abstract

**Objective:**

To explore the association between health behaviors and habits of university students and academic achievement.

**Participants:**

Six hundred fourteen undergraduate students at a state university in the United States.

**Methods:**

Students were invited over a 2-year period to participate in an anonymous online survey that asked questions concerning a wide range of health behaviors and habits; participants were asked to report their current grade point average (GPA). Standard Least Squares Models were used to examine differences in self-reported GPA across the different health behaviors and habits, with individuals as replicates.

**Results:**

The study found positive associations between breakfast consumption, physical activity, and strength training and self-reported GPA, and negative associations between the hours of sleep per night, hours worked per week, fast food and energy drinks consumption, and use of marijuana, alcohol and electronic vaping products.

**Conclusions:**

While there is an association for some of the studied health behaviors and habits with self-reported GPA, the effect sizes for these health behaviors were low. The significant effect of vaping on GPA as well as the increased use reported in this study indicates that the topic should be explored further. Furthermore, students should be educated on the potential positive and negative effects of health behavior choices to help them make better choices.

## Introduction

While the terms ‘academic performance’ and ‘academic success’ are widely used, there is no easy or universally acknowledged definition of either term (York, Gibson & Rankin, 2015). In the United States, and many other countries, grades earned and a grade point average (GPA) calculated from these grades are used as measures of academic success (Şahin, Çekin & Yazıcılar Özçelik, 2018). Looking into factors that may impact college students’ academic success in a positive or negative way is important because the information gained can be used to educate students about these factors to enable them to make choices that minimize negative choices and maximize positive choices.

Although adherence to appropriate health behaviors can assist in facilitating an overall healthy lifestyle in adulthood, the transition to college is often accompanied by an increase in unhealthy behaviors that may influence students’ academic performance (Ruthig et al., 2011; Office of Disease Prevention & Health Promotion, 2020). Published studies have shown that students who do follow public health recommendations for their lifestyle choices achieve higher GPAs (Trockel, Barnes & Egget, 2000; Wald et al., 2014). Health-promoting behaviors, such as consumption of fruits and vegetables, regular sleep routines, adequate physical activity, and frequent breakfast intake are positive predictors of GPA (Bellar et al., 2014; Wald et al., 2014; Hershner, 2020; Reuter, Forster & Brister, 2020). On the contrary, unhealthy behaviors such as smoking or vaping, use of alcohol or drugs, consumption of fast food, and working long hours have been identified as negative predictors of academic performance (Tessema, Ready & Astani, 2014; Arria et al., 2015; Meda et al., 2017; Lee, Baring & Sta. Maria, 2018; Reuter, Forster & Brister, 2020).

Sleep is not only a necessity for biological functioning, but is a vital component for maintaining cognitive roles, memory consolidation, decision making, and learning in general (Hershner, 2020). Sleep quality and quantity are among the most researched health behaviors in connection with academic performance of university students, and there is a consensus that both can affect students’ grades (Gilbert & Weaver, 2010; Flueckiger et al., 2014; Gaultney, 2016; Gomez Fonseca & Genzel, 2020). In Gaultney’s study, for example, freshmen at risk for a sleeping disorder, or infrequent restful sleep, did receive lower grades (Gaultney, 2016). Chen & Chen (2019) found that four out of ten college freshmen reported chronic sleep deprivation, and furthermore, that sleep deprivation in general was associated with a lower GPA.

Because of the rising costs of attending university as well as covering basic expenses while enrolled, there has been a sharp increase in the number of students relying on income from working while in college (National Center for Education Statistics, 2020). Zhang, Shao Tarleton & Johnston (2019) found that seven out of ten students were working for the duration of the semester, while almost half were averaging over 20 h a week. Other studies reported that students who worked ten or less hours over the course of the week reported the highest GPAs, while working more than 10 h was linked with lower GPAs (Tessema, Ready & Astani, 2014; Andemariam et al., 2015).

Although a less studied topic relating to university students’ academic performance, physical activity may have a positive effect on overall GPA. According to the Committee on Physical Activity and Physical Education in the School Environment of the Institute of Medicine, basic cognitive functions related to attention and memory are enhanced by physical activity (Kohl & Cook, 2013). Bellar et al. (2014) reported that higher levels of physical activity are positively associated with a higher GPA, while Roddy, Phle-Krauza & Geltz (2017) reported that more frequent use of a university’s recreation center was associated with higher GPAs. On the other hand, Flueckiger et al. (2014) did not find a clear correlation of physical activity with increased learning goal achievement or exam grades.

Healthy eating habits have been shown to positively influence academic performance (Kristjánsson, Sigfúsdóttir & Allegrante, 2010; Wald et al., 2014; Burrows et al., 2017). The quality of students’ diet appears to be the main factor for this effect. Florence, Asbridge & Veugelers (2008) reported an association of diet quality and academic performance in school children. They found that students with decreased overall diet quality were significantly more likely to perform poorly on structured assessments. Rampersaud et al. (2005) also expressed that a healthy diet is effective in improving cognitive functioning and academic performance. Not all studies, however, agree on which habits specifically have a positive effect on academic performance. For example, Burrows et al. (2017) looked at seven different studies and found that five of them reported higher academic achievement with increased fruit intake. In contrast, Trockel, Barnes & Egget (2000) and Reuter, Forster & Brister (2020) did not report an association between fruit intake and GPA. Regular consumption of breakfast has also shown to have a positive effect on GPA (Chawla et al., 2019; Reuter, Forster & Brister, 2020). Conversely, unhealthy dietary behaviors adversely affect academic performance. Kobayashi (2009) and Reuter, Forster & Brister (2020) found that as consumption of fast food increased, GPA successively decreased, and consumption of energy drinks also has shown to have a negative effect on academic success (Trunzo et al., 2014; Champlin, Pasch & Perry, 2016).

The two most commonly abused substances among college students are alcohol and marijuana (Meda et al., 2017). Both have been shown to be related to decreased academic performance in a number of published studies (Wallis et al., 2019). Drug use is considered a major factor associated with inferior academic success and more frequent alcohol consumption is negatively correlated with GPA (Arria et al., 2015; Conway & DiPlacido, 2015; Meda et al., 2017; Souza, Hamilton & Wright, 2019). Marijuana use was found to be negatively correlated with GPA, with more frequent users having the lowest GPAs (Suerken et al., 2016).

Limited studies exist concerning cigarette smoking and e-cigarette use, and their relationship with college students’ GPA. Lee, Baring & Sta. Maria (2018) reported lower GPAs in students who smoked compared to their peers who did not. Phillips et al. (2015) also found that smoking was a negative predictor of cumulative GPA. The use of e-cigarettes or other electronic vaping products is a fairly new phenomenon and, thus, there are only a few published studies on their use among university students and possible effects on academic performance. Onojighofia Tobore (2019) stressed that induced oxidative stress from e-cigarettes can be linked to cognitive impairment and attention deficit. Leas et al. (2020) reported that almost one-third of the young adults they surveyed had used at least one form of tobacco product, including e-cigarettes. However, the study did not look into an association with academic performance.

Finally, there is a long history of university students turning towards the use of stimulants to improve academic performance (Arria & DuPont, 2010). However, Arria et al. (2015) reported that users of stimulants and analgesics had lower average GPAs than nonusers. The students also spent less time studying, went out more socially, and skipped class more frequently.

This article reports on 28 health behaviors and habits and their impact on the academic performance of undergraduate students at a public university in the southern United States. The behaviors selected can be categorized in five categories: (1) sleeping habits, (2) working, (3) physical activity, (4) eating habits, and (5) alcohol, tobacco, and drugs consumption (Table 1). Our aim was to cover the spectrum of behaviors and habits customarily found in a student population. Therefore, we included basic behaviors, such as sleeping and eating habits, as well as behaviors and habits that only some students may engage in, such as alcohol consumption.

**Table 1:** Relationship between different types of behaviors and habits among university students and self-reported current GPA. Standard Least Squares Model (Restricted Maximum Likelihood Method, REML; individuals as replicates; students’ biological sex used as a random effect).

Behaviors and Habits	Independent variable	Sample size	Test Results	*p*-value
Sleeping habits				
Hours of sleep per night	Average number of hours slept per night (categorical data: 4 h or less; 5 h; 6 h; 7 h; 8 h; 9 h; 10 h or more per night)	614	DF/DFDen = 6/605.2, F-Ratio = 4.4473, R^2^ = 0.050	0.0002*
Average time going to bed	Average time of day going to bed (categorical data: before 8 pm; between 8 pm and 10 pm; between 10 pm and midnight; between midnight and 2 am; after 2 am)	614	DF/DFDen = 4/606.2, F-Ratio = 2.5648, R^2^ = 0.024	0.0373
Average wake up time	Average time of day waking up (categorical data: before 6 am; between 6 am and 8 am; between 8 am and 10 am; between 10 am and noon; after noon)	614	DF/DFDen = 4/604.1, F-Ratio = 1.0075, R^2^ = 0.015	0.4029
**Working**				
Do you work?	Categorical data (Yes; No)	614	DF/DFDen = 1/271.5, F-Ratio = 6.8855, R^2^ = 0.018	0.0092*
Average hours worked per week	Average number of hours worked per week (categorical data: [including all students who did not work]; under 5 h; 5-10 h; 10–20 h; 20–30 h; 30–40 h; over 40 h)	614	DF/DFDen = 6/565.8, F-Ratio = 3.3642, R^2^ = 0.041	0.0029*
**Physical activity**				
Physical activity (aerobic exercise)	Number of days with physical activity of at least 60 min per week (continuous numerical data, range: 0 to 7 days)	605	DF/DFDen = 1/597.7, F-Ratio = 5.5449, R^2^ = 0.019	0.0189*
Strength training	Number of days per week doing exercises to strengthen or tone muscles (continuous numerical data, range: 0 to 7 days)	605	DF/DFDen = 1/599, F-Ratio = 7.9203, R^2^ = 0.023	0.0050*
**Eating habits**				
Consumption of vegetables	Number of times vegetables consumed in past 7 days (categorical data: 0 times; 1 to 3 times; 4 to 6 times; 7 to 10 times; 11 times or more)	614	DF/DFDen = 4/601.9, F-Ratio = 1.1902, R^2^ = 0.015	0.3139
Consumption of fruit	Number of times fruit consumed in past 7 days (categorical data: 0 times; 1 to 3 times; 4 to 6 times; 7 to 10 times; 11 times or more)	614	DF/DFDen = 4/607, F-Ratio = 2.4016, R^2^ = 0.023	0.0488
Consumption of 100% fruit juice	Number of times 100% fruit juice consumed in past 7 days (categorical data: 0 times; 1 to 3 times; 4 to 6 times; 7 to 10 times; 11 times or more)	614	DF/DFDen = 4/605.6, F-Ratio = 1.4007, R^2^ = 0.017	0.2322
Consumption of green salad	Number of times green salad consumed in past 7 days (categorical data: 0 times; 1 to 3 times; 4 to 6 times; 7 to 10 times; 11 times or more)	614	DF/DFDen = 4/595.3, F-Ratio = 1.3806, R^2^ = 0.017	0.2392
Consumption of milk	Number of glasses of milk consumed in past 7 days (categorical data: 0 times; 1 to 3 times; 4 to 6 times; 7 to 10 times; 11 times or more)	614	DF/DFDen = 4/604.7, F-Ratio = 1.9403, R^2^ = 0.022	0.1022
Breakfast consumption	Number of days breakfast eaten in past 7 days (continuous numerical data, range: 0 to 7 days)	614	DF/DFDen = 1/611.5, F-Ratio = 34.14167, R^2^ = 0.062	<0.0001*
Consumption of soda products	Number of times soda consumed in past 7 days (categorical data: 0 times; 1 to 3 times; 4 to 6 times; 7 to 10 times; 11 times or more)	614	DF/DFDen = 4/607.1, F-Ratio = 1.3341, R^2^ = 0.016	0.2558
Consumption of diet soda products	Number of times diet soda consumed in past 7 days (categorical data: 0 times; 1 to 3 times; 4 to 6 times; 7 to 10 times; 11 times or more)	614	DF/DFDen = 4/607, F-Ratio = 1.4606, R^2^ = 0.017	0.2127
Consumption of energy drinks	Number of times energy drinks consumed in past 7 days (categorical data: 0 times; 1 to 3 times; 4 to 6 times; 7 to 10 times; 11 times or more)	614	DF/DFDen = 4/607.2, F-Ratio = 3.4538, R^2^ = 0.028	0.0084*
Consumption of sports drinks	Number of times sports drinks consumed in past 7 days (categorical data: 0 times; 1 to 3 times; 4 to 6 times; 7 to 10 times; 11 times or more)	614	DF/DFDen = 4/585.2, F-Ratio = 0.5244, R^2^ = 0.012	0.7179
Fast food consumption	Number of times fast food consumed in past 7 days (categorical data: 0 times; 1 to 3 times; 4 to 6 times; 7 to 10 times; 11 times or more)	614	DF/DFDen = 4/574, F-Ratio = 5.5176, R^2^ = 0.040	0.0002*
**Alcohol, smoking, and drugs consumption**				
Have you ever tried cigarette smoking?	Categorical data (Yes; No)	614	DF/DFDen = 1/611.5, F-Ratio = 10.1090, R^2^ = 0.025	0.0016*
Do you consider yourself to be a smoker?	Categorical data (Yes; No)	598	DF/DFDen = 1/595.9, F-Ratio = 0.0044, R^2^ = 0.010	0.9470
Have you ever used an electronic vaping product?	Categorical data (Yes; No)	614	DF/DFDen = 1/609.5, F-Ratio = 8.7670, R^2^ = 0.022	0.0032*
Vaping frequency	Number of days electronic vaping product used in the past 7 days (continuous numerical data, range: 0 to 7 days)	597	DF/DFDen = 1/587.4, F-Ratio = 0.6937, R^2^ = 0.018	0.6775
Have you ever consumed alcoholic drinks?	Categorical data (Yes; No)	614	DF/DFDen = 1/607.4, F-Ratio = 9.5925, R^2^ = 0.024	0.0020*
Alcohol consumption	Number of days alcoholic beverages consumed in the past 30 days (categorical data: 0 days; 1 or 2 days; 3 to 5 days; 6 to 9 days; 10 to 19 days; 20 to 29 days)	614	DF/DFDen = 5/605.6, F-Ratio = 0.3222, R^2^ = 0.011	0.8997
Have you ever used marijuana?	Categorical data (Yes; No)	614	DF/DFDen = 1/611.2, F-Ratio = 14.4449, R^2^ = 0.030	0.0002*
Marijuana use frequency	Number of days marijuana used in the past 7 days (continuous numerical data, range: 0 to 7 days)	614	DF/DFDen = 1/610.2, F-Ratio = 2.9095, R^2^ = 0.012	0.0886
Pain killer abuse	Number of times prescription pain medication taken without prescription or differently than prescribed (categorical data: 0 times; 1 or 2 times; 3 to 9 times; 10 to 19 times; 20 to 39 times; 40 or more times)	614	DF/DFDen = 5/606.1, F-Ratio = 2.2810, R^2^ = 0.03	0.0453
Other drug use	Have you ever used any drugs other than marijuana? (list of examples provided) (categorical data; Yes; No)	614	DF/DFDen = 1/611.9, F-Ratio = 0.6341, R^2^ = 0.009	0.4262

**Notes:**

* Significant using the Benjamini–Hochberg Procedure.

The last study to look at a similar, although different number of factors in a comparable study population was published in 2000 (Trockel, Barnes & Egget, 2000). The students in that study were late Generation X and/or early Millennial students; the students in our study are Generation Z students whose health behaviors and habits are different in many ways (Rue, 2018; Schlee, Evelan & Harich, 2020). For example, vaping was not prevalent in 2000, while smoking was far more common. For this reason, the findings of this study are of particular importance because of gaps in our understanding of health-promoting behaviors and the academic performance of this new generation of college students.

## Methods

### Ethical research statement

The research protocol and its amendment were approved by an ethical review board (Institutional Review Board) at Florida Gulf Coast University (FGCU) prior to data collection (FGCU IRB 2018-17, March 30, 2018). All researchers involved in the study were trained in ethical data collection through the Collaborative Institutional Training Initiative (CITI). Data collection followed all laws relevant to the survey of university student populations.

### Data collection

Data were collected over a 2-year period between April 1, 2018 and January 31, 2020 using an anonymous online survey. Students at all colleges at a regional state university in southwest Florida were invited via email to participate in the survey. The first page of the survey consisted of an approved online survey consent form; in other words, consent was obtained. Participation was voluntary and participants did not receive any compensation or extra credit. Some part of the data unrelated to current research purpose was published elsewhere (Reuter, Forster & Brister, 2020).

The survey consisted of five groups of questions around health and wellness, requesting information–among other topics–regarding demographic information, such as gender, age, ethnicity/race, year at school, and current overall grade point average (GPA), eating and sleeping habits, and drugs and alcohol consumption (see Appendix 1). Most of the questions were modeled after questions used in the 2017 Standard High School Youth Risk Behavior Survey (Centers for Disease Control & Prevention, 2017).

### Data analyses

For questions with categorical answers, data are presented as a percentage of the total participant pool, or a portion of this pool. For questions with quantitative answers, data are presented as means with standard deviations. Sample sizes are indicated as they vary for different analyses due to the voluntary nature of the survey.

To examine differences in self-reported GPA across 28 different health behaviors and habits, Standard Least Squares Models were used (Restricted Maximum Likelihood Method, REML), with individuals as replicates. GPA differed significantly by cohort (Wilcoxon Rank Sums Test, Chi-square = 18.9057, DF = 3, *p* = 0.0003) and was therefore included as a random effect in all tests. GPA differed by biological sex (Wilcoxon Rank Sums Test, Chi-square = 4.5894, DF = 1, *p* = 0.0322) and biological sex of students was therefore included as a random effect in all tests.

Student’s T-Test All Pairwise Comparisons (Least Squares Means) were used as post-hoc tests. The dependent variable in all tests was student self-reported GPA (a continuous numerical variable ranges from 0.00 to 4.00). Independent variables are listed in Table 1 below or can be found on the survey in Appendix 1. Given the number of statistical analyses performed (41 tests) we have used the Benjamini–Hochberg Procedure (with a false discovery rate of 5%) to determine significance (tests with a *p*-value < 0.0189 are being considered significant using this approach). Statistical analyses were performed using JMP software program Version 15 (JMP^®^; SAS Institute Inc., Cary, NC, USA).

## Results

### Study population

Of 761 students who participated in the online survey, 147 respondents were excluded from analyses for a number of reasons, including: (a) respondents who indicated an age younger than 18 years of age or older than 25 years of age or failed to provide information on their current GPA; (b) part-time students, as the sample sizes was too small (*n* = 15) and they had a lower GPA than full-time students (Wilcoxon Rank Sums Test, Chi-square = 4.61, DF = 1, *p* = 0.0317); (c) non-traditional students (graduate, non-degree seeking, and second-degree seeking students); and (d) students who failed to provide information about their biological sex. As such, 614 students remained in the dataset.

**Study population profile**

**Biological sex**: 78.5% female students, 21.5% male students.

**Race/ethnicity**: 58.3% Caucasian/White, 15.8% Hispanic, 5.9% African-American/Black, 1.1% East Asian, 1.1% Non-Hispanic, 17.3% more than one ethnicity/race or ethnicity/race other than listed, 0.5% no information.

**Age**: 19.6 ± 1.4 years (mean ± standard deviation; range: 18–25 years; median age = 19 years).

**Student population**: 30.9% freshman, 29.5% sophomore, 26.6% junior, 13.0% senior.

The average current GPA for all 614 respondents was 3.39 ± 0.51 (range: 0.40–4.0; median GPA = 3.5). Freshmen reported the highest average GPA (3.45 ± 0.64), followed by seniors (3.45 ± 0.42), juniors (3.36 ± 0.39), and sophomores (3.36 ± 0.48).

The average GPA for female students was 3.42 ± 0.52 compared with 3.34 ± 0.49 for male students.

### Health behaviors and self-reported GPA

Differences in self-reported GPA across 28 different health behaviors and habits were examined (Table 1).

### Sleep

**Figure 1:**
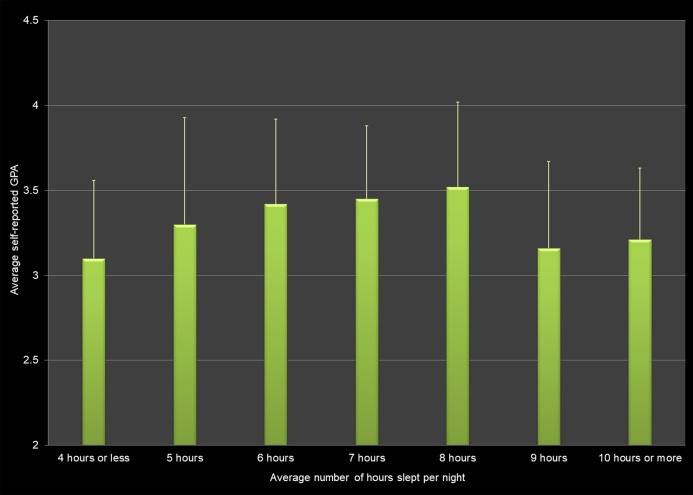
Average number of hours slept per night and average self-reported grade point average (GPA) across all respondents.

Average self-reported GPA differed by the number of hours slept on average per night (Fig. 1). Students who slept 4 h or less per night had lower GPAs than students who slept 6 h per night (*p*-value = 0.0062), 7 h per night (*p*-value = 0.0025), and 8 h per night (*p*-value = 0.0004, Student’s T-Test All Pairwise Comparisons). Students who slept 9 h per night, had lower GPAs than students who slept 8 h per night (*p* = 0.0006, Student’s T-Test All Pairwise Comparisons).

There was no significant difference in the average GPA of students based on when they went to sleep. Nine out of ten respondents went to bed between 10 pm and 2 am (87.3%). Students going to bed between 8 pm and 10 pm (4.1%) had a self-reported GPA of 3.46 (±0.33), students going to bed between 10 pm and midnight (44.1%) reported an average GPA of 3.45 (±0.47), and the average GPA for students who went to bed between midnight and 2 am (43.2%) was 3.37 (±0.53).

Almost equal numbers of students got up before 6 am (8.5%) or liked to sleep in and got up after 10 am on average (8.6%). The rest preferred to get up between 6 am and 8 am (42.0%) or between 8 am and 10 am (40.9%). But, there was no significant difference in the average GPA of students based on when they woke up.

### Working

**Figure 2:**
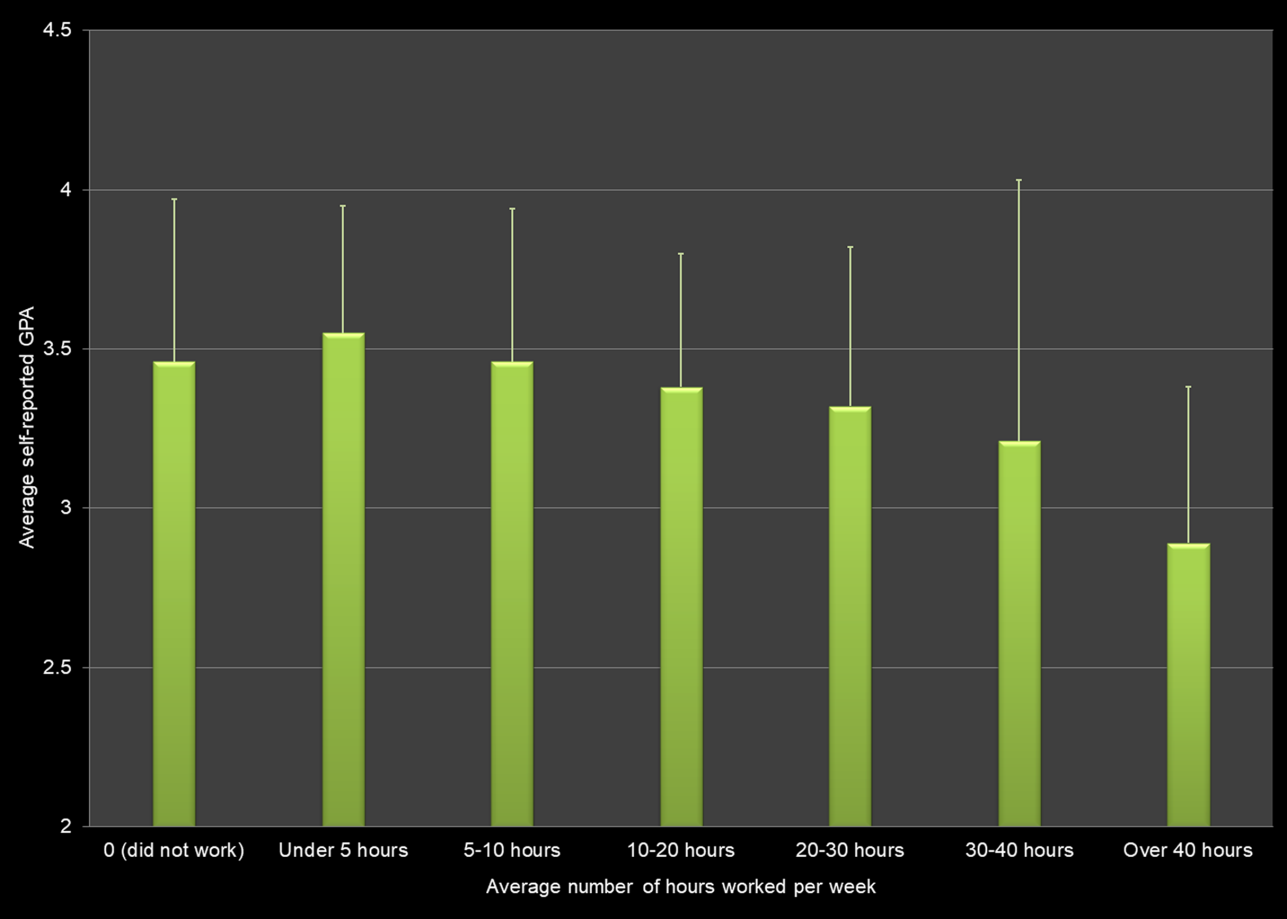
Average number of hours worked per week and average self-reported grade point average (GPA) across all respondents.

Self-reported GPA for respondents differed depending on whether or not respondents worked, with a negative association between hours worked on average per week and self-reported GPA. Students who did not work/worked zero hours per week had a higher GPA than students who worked 20–30 h per week (*p* = 0.0188), 30–40 h per week (*p* = 0.0094), and more than 40 h per week (*p* = 0.0014). Students who did not work or only up to 10 h per week reported average GPAs of above 3.45, whereas students who worked 10 h or more per week had GPAs of 3.38 or less (Fig. 2).

### Physical activity

One-sixth of respondents considered themselves as not physically active at all (17.0%). Their self-reported GPA was 3.28 ± 0.64 as compared to an average GPA of 3.43 ± 0.48 for respondents who were physically active (83.0%). There was a significant difference in the average GPA of students based on the number of days they were physically active in the week prior.

In addition, respondents who engaged in exercises to strengthen or tone their muscles, such as push-ups, sit-ups, or weight lifting, reported higher GPAs than students who did not engage in such exercises.

### Eating habits

Consumption habits for vegetables, fruits, fruit juice, or green salad did not change among students with different GPAs. There was also no significant difference in the average GPA of students based on the number of times milk, soda, diet soda, or sports drinks were consumed in the last week either. Nevertheless, the difference in average GPA between respondents who did and those who did not consume energy drinks was significant with a *p*-value of 0.0084.

**Figure 3:**
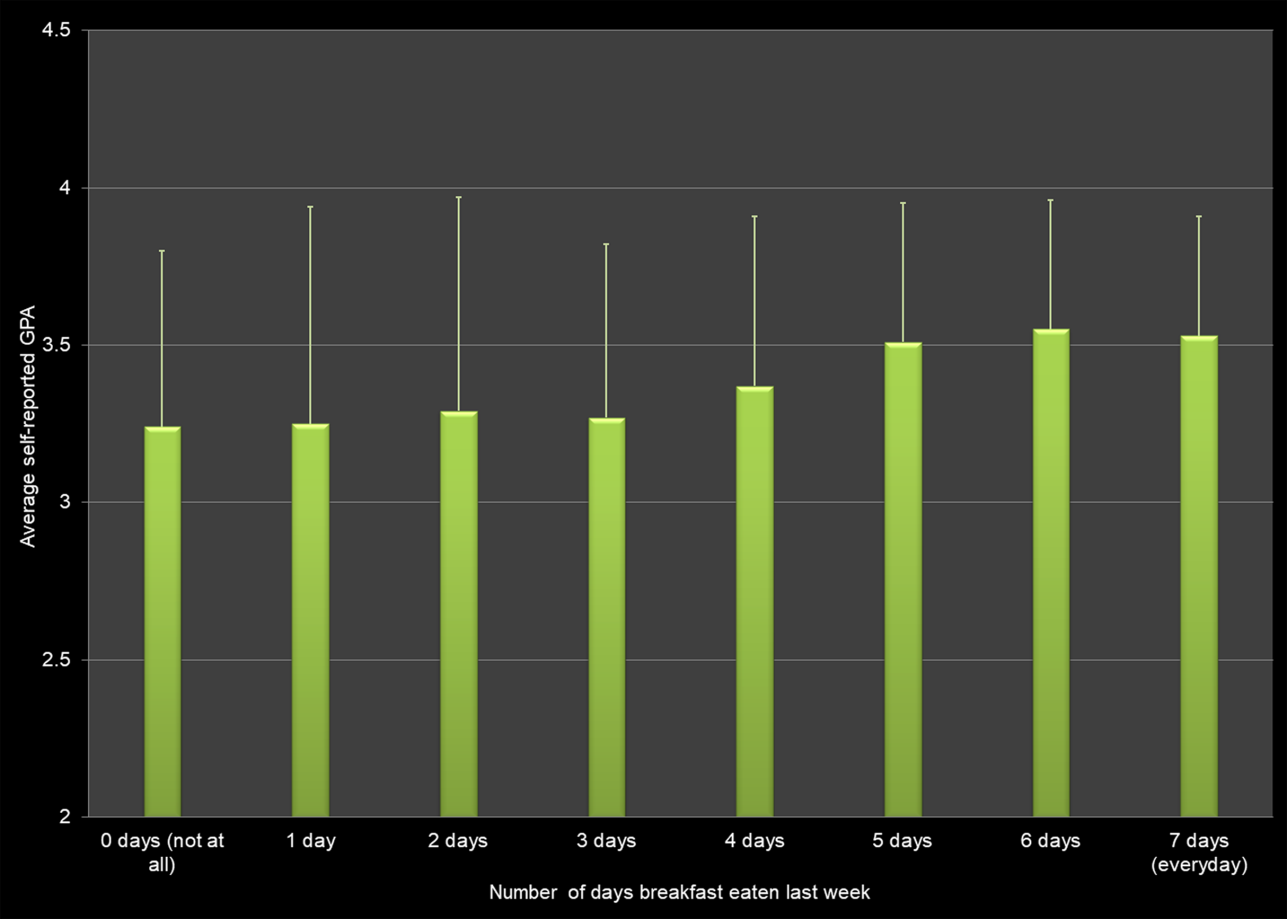
Frequency of eating breakfast (measured in number of days that breakfast was eaten in the last week) and average self-reported grade point average (GPA) across all respondents.

Self-reported GPA differed based on the days that students had eaten breakfast in the past seven days (Fig. 3). Students who ate breakfast seven days a week/everyday were more likely to have higher GPAs than students who never ate breakfast (zero days of the last seven), ate breakfast one day per week, ate breakfast 2 days per week, or ate breakfast three days per week (*p* < 0.0075).

**Figure 4:**
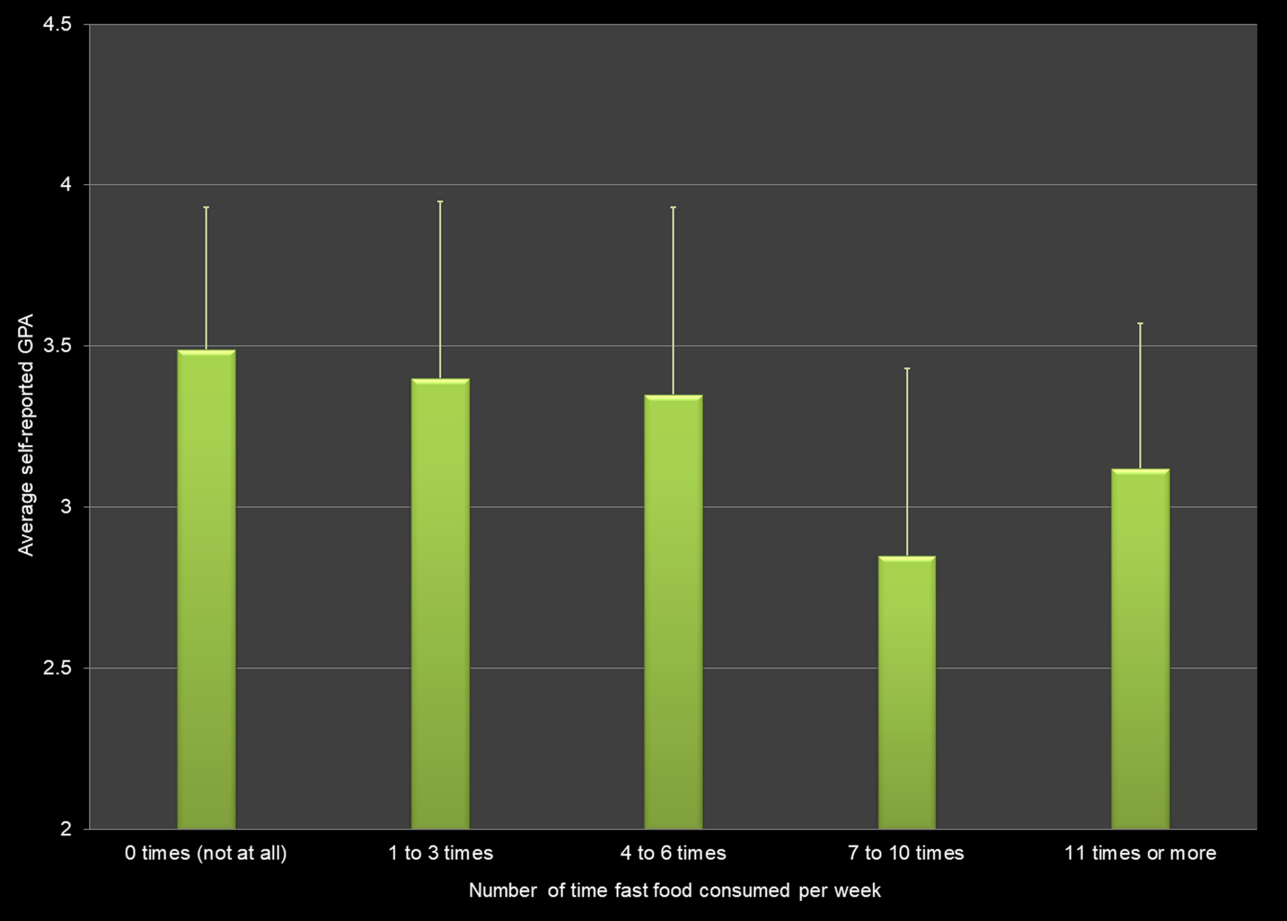
Fast food consumption (measured as how often fast food was eaten in the last week) and average self-reported grade point average (GPA) across all respondents.

Self-reported GPA differed based on the number of times that students had eaten fast food in the past 7 days (Fig. 4). Students who ate no fast food had higher GPAs than students who reported eating fast food 7 to 10 times (*p* < 0.0001).

### Alcohol, smoking, and drugs consumption

The self-reported GPAs for students who had consumed alcoholic drinks, students who had tried cigarette smoking, and students who had used electronic vaping products were lower versus students who had not engaged in any of these behaviors before.

There was also a significant difference in self-reported GPA of students based on whether or not they had ever used marijuana at all, with students who had never used marijuana reporting higher GPAs than those who had used it at least once.

Conversely, there was no significant difference in the average GPA of students based on whether or not they considered themselves to be a smoker, the number of times they had used an electronic vaping product in the last week, the number of alcoholic beverages they had consumed in the last 30 days, and the number of times they had used marijuana in the past 7 days.

There was also no significant difference in the average GPA of students based whether or not they had ever taken prescription pain medication without a prescription or differently than prescribed, and whether or not they had ever consumed drugs other than marijuana.

The most commonly named drugs were hallucinogens (*n* = 40) such as LSD and DMT, cocaine (*n* = 34), ecstasy/molly/MDA (*n* = 24), and mushrooms (*n* = 17). Surprisingly, Xanax^®^ and ADHD medications, such as Adderall^®^, were listed by five respondents each only, although they are reportedly used more frequently (National Survey on Drug Use & Health, 2017).

## Discussion

The findings of this study are of particular importance because they fill in gaps of our understanding of health behaviors and their impact on the academic performance of Generation Z university students within a university in the southern United States of America. Given, however, that health behaviors and habits vary considerably within a region or country, and from university to university, it is nevertheless difficult to compare our results to the behavior of student populations at other institutions. For example, a study comparing health promoting behaviors and lifestyle characteristics of students in the United Kingdom found marked differences for the students at the seven universities included (El Ansari et al., 2011). Trockel, Barnes & Egget (2000) acknowledged that some of their findings would probably have been different had they not collected data at a ‘dry’ campus, i.e., a campus where alcohol consumption was prohibited. Also, the arrival of Generation Z students (i.e., students born after 1996) on campus over the last 5 to 6 years has brought changes that make it more difficult to compare our results with the findings of studies published 15 or 20 years ago. For instance, according to the American Lung Association (2020), in 1999, 38.4% of high school students were smokers; by 2018, that percentage had dropped down to 8.8%. Still, the results of our study align with previously published results.

### Sleep

Our findings that students who sleep less than 4 h or more than 9 h per night have lower GPAs than students who sleep 6, 7, or 8 h per night, are similar to what Vedaa et al. (2019) reported for Norwegian students. Chen & Chen (2019) reported that a lack of sleep has a negative association with GPA for college freshmen.

However, our data did not confirm the results from other studies that indicated the timing of sleep to be a better predictor for academic performance than actual hours slept (Eliasson, Lettieri & Eliasson, 2010; Genzel et al., 2013). For example, Eliasson, Lettieri & Eliasson (2010) reported that students with earlier bed times and earlier wake times had higher GPAs, and concluded that timing of sleep is a better predictor for academic performance than actual sleep hours. In our study, however, it did not matter what time students went to bed as long as they got a sufficient amount of sleep. For instance, students who went to bed between midnight and 2 am reported an average GPA of 3.24 ± 0.60 if they slept 5 h or less but an average GPA of 3.47 ± 0.60 if they slept 8 h per night.

### Working

There is a consensus among published studies that the number of hours that full-time students work negatively affects their academic performance (Miller, Danner & Staten, 2008; Pike, Kuh & Massa-McKinley, 2008; Darolia, 2014; Tessema, Ready & Astani, 2014; Andemariam et al., 2015). In line with our study, Tessema, Ready & Astani (2014) reported lower GPAs for students working more than 11 h per week, and students working over 31 h per week had the lowest GPA in the study by Andemariam et al. (2015).

Additionally, the positive effect on average GPA we found for students working fewer than 10 h per week (Fig. 2), is supported by the studies of Darolia (2014), Andemariam et al. (2015), and Tessema, Ready & Astani (2014).

### Physical activity

We found a positive effect for both strength training and physical activity in general on average self-reported GPA. Respondents who engaged in exercises to strengthen or tone their muscles, such as push-ups, sit-ups, or weight lifting, on four days or more during the past seven days had a higher GPA at 3.51 ± 0.44 than students exercising 1-3 days (3.43 ± 0.44) and students who did not exercise at all (3.28 ± 0.64).

However, Wald et al. (2014) reported a modestly higher GPA for physical activity only and no association between GPA and strength training. Trockel, Barnes & Egget (2000) did not find an association between GPA and either exercise or strength training; nor did Flueckiger et al. (2014). Contrary to that, Bellar et al. (2014) reported that higher levels of physical activity are positively associated with higher GPAs. Roddy, Phle-Krauza & Geltz (2017) showed that students who used a university’s recreation center frequently and regularly were the students with the higher GPAs as compared to their peers who visited irregularly. However, they did not look at the different types of exercise and how they affected GPA.

### Eating habits

We found a positive association between eating breakfast and GPA as well as a negative association between the number of times fast food was consumed per week and GPA. The positive effect of eating breakfast regularly has been reported in studies from different countries (Trockel, Barnes & Egget, 2000; Phillips, 2005; Rampersaud et al., 2005; Adolphus, Lawton & Dye, 2013; Chawla et al., 2019; Reuter, Forster & Brister, 2020). Kobayashi (2009), Deliens et al. (2013) and Reuter, Forster & Brister (2020) also found a negative association between fast food consumption and academic performance.

In agreement with previous studies (Petit & DeBarr, 2011; Trunzo et al., 2014; Buchanan & Ickes, 2015; Champlin, Pasch & Perry, 2016), our study showed a significant negative association between energy drink consumption and GPA. Interestingly, 84.3% of our respondents reported not having consumed energy drinks at all over the past seven days. On the other hand, Petit & DeBarr (2011) reported that more than half the students in their study drank at least one energy drink in the past seven days, illustrating how this health behavior has changed over the last 10 years.

### Alcohol, smoking, and drugs consumption

A negative relationship between alcohol consumption and GPA was reported by Conway & DiPlacido (2015), Piazza-Gardner, Barry & Merianos (2016) as well as Meda et al. (2017). However, we did not find an association between the number of alcoholic beverages consumed in the past 30 days and GPA. But, students in our study who indicated having consumed alcohol at least once so far reported lower GPAs than students who had never had an alcoholic drink.

Smoking clearly is not as popular with current college students as it used to be. Only one-fifth (19.5%) of students who answered the question “*Have you ever tried cigarette smoking?*” answered ‘yes’ and only 10 students (1.7%) considered themselves to be smokers. Still, students who had tried cigarette smoking reported lower GPAs than students who had never smoked. Phillips et al. (2015) and Meda et al. (2017) also reported that smoking was a negative predictor of cumulative GPA.

Vaping was much more common than smoking among our study population; one-third of respondents (35.9%) answered ‘yes’ to the question “*Have you ever used an electronic vapor product?*” and 15.1% of respondents had vaped during at least 1 day over the last 7 days. The fact that in our study only 22.5% of seniors but 39.5% of freshmen admitted to having used electronic vaping products demonstrates that vaping is still on the rise. Study respondents who reported having vaped before had lower GPAs than respondents who had never vaped.

The only drug used by a substantial number of respondents in this study was marijuana with 44.2% of participants responding with ‘yes’ to the question “*Have you ever used marijuana?*”. While we did not find an association between marijuana use frequency (i.e., number of days marijuana used in the past 7 days) and GPA, our data show that students who answered the question with ‘yes’ reported lower GPAs compared with students who answered with ‘no’. Arria et al. (2015) did not find an association between marijuana use and GPA, whereas Meda et al. (2017), Suerken et al. (2016), Souza, Hamilton & Wright (2019), and Wallis et al. (2019) all reported a negative relationship between marijuana use and academic success.

We did not find an association between pain killer abuse (i.e., taking prescription pain medication without prescription or differently than prescribed) or the use of drugs other than marijuana, such as cocaine, heroin, methamphetamines, ecstasy, hallucinogenic drugs, or synthetic marijuana, and GPA. Pain killer abuse among our study population was at about the same level as reported for the age group 18–25 in the 2017 National Survey on Drug Use and Health (NSDUH) at 7.7% in our study vs. 7.2% in the NSDUH.

The three main limitations of our study were: (1) participant selection, (2) reliance on self-reported health behaviors and habits, and (3) reliance on self-reported GPAs. Although we invited students from all colleges across the university to participate, our final study population with 78.5% female students and 21.5% male students does not reflect the demographics of the student body at our university (53% female students and 47% male students) (Florida Gulf Coast University, 2020). Research has shown that female students are more interested in or worried about health-related issues and diet, and are more likely to participate in online surveys in general (Rozin, Bauer & Catanese, 2003; Smith, 2008).

Participants may have intentionally or unintentionally provided incorrect information about their lifestyle choices or their current GPA. For example, students may have given us the GPA they were hoping to achieve at end of the current semester instead of the actual GPA for completed classes. Also, students may have wanted to appear to be making better health behavior choices or may not remember accurately how often they had consumed fast food, for example. Moreover, some of the behaviors and habits included in the survey were not defined and participants may have interpreted them differently. For example, some participants may have considered walking to class as being physically active, while others may have set the bar higher. Additionally, we cannot rule out that students may have participated more than once. However, there is no reason to believe that inaccuracies such as these or repeat responses had a substantial impact on the findings of this study due to the number of participants being higher than 600.

Our survey was not designed to look into factors that affect student health behaviors and habits; it was designed to look at whether or not the included behaviors impact students’ academic performance. Therefore, the information received from participants is not suitable to make inferences as to potential mechanisms underlying the findings of our study. These questions should be addressed in future studies.

## Conclusions

Our study involving more than 600 students found positive associations between breakfast consumption, physical activity, and strength training and self-reported grade point average. We also found negative associations between the hours of sleep per night, hours worked per week, fast food and energy drinks consumption, and use of marijuana, alcohol and electronic vaping products. However, the effect sizes for these health behaviors were low. In other words, even though there is a relationship to the average GPA, the variables do not explain the variation in GPA scores. For example, although self-reported GPA differs significantly by hours of sleep per night, only 5.0% of the variation in the GPA scores can be explained by this factor alone.

So far, the public—as well as the research community—has focused mainly on the physical damage using electronic vaping products causes, such as lung injuries. The significantly negative effect of vaping on GPA as well as the increased use found in our study indicates that the topic should be explored in further studies.

## Supplemental Information

10.7717/peerj.11107/supp-1 Supplemental Information 1 Survey Student Health Behavior and Academic Success.

10.7717/peerj.11107/supp-2 Supplemental Information 2 Survey data.

## Additional Information and Declarations

### Data Availability

The following information was supplied regarding data availability: Raw data are available in the Supplemental Files.
