# Supplementary material for: Student health behavior and academic performance
Source: PeerJ. 2021 Apr 16;9:e11107. doi: 10.7717/peerj.11107 (PMC8054760; doi:10.7717/peerj.11107)
Supplement: Supplemental Information 1 [file peerj-09-11107-s001.pdf]

Student Health Behavior and Academic Success

Please take your time to read the following information carefully before starting the survey.

Online Survey Consent Form

Study Title: Student Health Behavior and Academic Performance

Principal Investigator: Dr. Peter Reuter

You are invited to participate in a research study conducted through Florida Gulf Coast University. You must be age 18 or older to participate in the study. Your participation in this study is voluntary. The University requires that we obtain your consent to participate in this study.

Refusal to join the study will not affect any future services you may be eligible to receive from the University.

Study Summary

The purpose of this study is to look at how health behavior impacts the academic success of college students. The information we learn from this study will increase overall knowledge and may help design better outreach programs at FGCU and other universities.

Participants in this study will be asked to complete an anonymous online survey. Completing the survey will take less than 20 minutes.

There is a very low risk of a potential invasion of privacy of the participants and/or their families as well as the social risk associated with revealing recreational drug use or prescription drug abuse.

If you are interested in learning more about this study, please continue to read below and contact me (preuter@fgcu.edu; 239-590-7512) with any questions you have to help you understand the study.

Your participation in the study is completely voluntary. If you decide to participate now you may change your mind and stop at any time, for any reason, without penalty or loss of any future services you may be eligible to receive from the University.

Purpose of the Study

The purpose of the study is to look at how health behavior impacts the academic success of college students. There is a limited number of studies that looked at some aspects of student health behavior and a possible correlation to academic success, however, none of them looked at the variety of factors our study will include.

Invitation to Participate in the Study

We are asking you to take part in the study because you are college student at FGCU and can help us gather data and information for our study.

Description of your Involvement

If you agree to be part of the research study, you will be asked to complete an anonymous online survey about your health behavior choices, such as eating and drinking habits, sleep, and use of recreational drugs or prescription drugs. We expect the survey will take less than 20 minutes to complete. The survey cannot be completed in more than one session.

Risks and Discomforts of Participation

The likelihood and seriousness of probing for personal or sensitive information in this survey is minimal. The same applies to a potential invasion of privacy of the participants and/or their families.

We consider the risks to be minimal because the survey is anonymous, participation is optional, and participants have the freedom to control the level of disclosure, i.e., skip questions or not answer any of them. However, participants may consider the risk to be higher than minimal because of questions about personal or sensitive information, such as past suicide attempts.

Your participation will be kept anonymous. However, working with email or the internet has the risk of compromising privacy, confidentiality, and/or anonymity. Despite this possibility, the risks to your physical, emotional, social, professional, or financial well-being are considered to be 'less than minimal' by completing the survey.

We understand that some participants may find it hard to answer certain questions or may get overwhelmed by emotions when describing events from the past.

If you are in immediate distress or crisis and need to speak to a clinician call the CAPS office and ask for an appointment or to speak with a clinician. Nights, weekends, and holidays, you may call the CAPS help line (239) 745-3277 and you will be connected to a clinician.

If you are imminent danger to harm yourself or others, call 911 if you are off campus and 590-1911 if you are on campus, or you may go to the emergency room at the nearest hospital.

You may also contact the National Suicide Prevention Lifeline at 1-800-273-TALK (8255).

Compensation for Participation

You will not be paid to take part in this study nor will you receive extra credit or any other form of compensation.

Confidentiality

If you join the study, we will make every effort keep your information confidential and secure by taking the following steps:

- The data will be collected anonymously via a Checkbox survey.
- Only the PI has access to the password-protected survey data.
- The data will be downloaded and stored on the PI's personal folder on the FGCU server as recommended by BTS.
- IP address will be stripped off before exporting the data out of Checkbox.

However, despite these safeguards, there is the possibility of hacking or other security breaches that could compromise the confidentiality of the information you provide. Thus, it is important to remember that you are free to decline to answer any question that makes you uncomfortable for any reason.

We will not release information about you unless you authorize us to do so or unless we are required to do so by law. If results of this study are published or presented at a professional meeting, no information will be included that would make it possible to identify you as a study participant.

It is possible that organizations responsible for making sure the research is done safely and properly such as the university, and government offices may need to see the information you provide.

Storage and Future Use of Data

The data will be stored on the PI's personal folder on the FGCU server. They can be destroyed be deleting the folder they are in.

We do not collect any paper records. However, should we print out surveys they will be destroyed using a professional shredding service no sooner than three years after completion of the research.

Voluntary Nature of Study

Participating in this study is completely voluntary. Refusal to join the study will not affect any future services you may be eligible to receive from the University. You can choose to not answer an individual question or you may skip any section of the survey by clicking “Next” at the bottom of the survey page to move to the next section.

If you choose to join the study, you can leave it at any time with no penalty.

Contact Information for the Study Team

We do not foresee any problems from participating in this study. However, if you believe you experienced a research related injury, please contact Dr. Peter Reuter at 239-590-7512 or via email (preuter@fgcu.edu).

If you have any questions about this study, please contact Dr. Peter Reuter at 239-590-7512 or via email (preuter@fgcu.edu).

Contact Information for Questions about Your Rights as a Research Participant

If you have any questions about your rights as a subject/participant in this research, or if you feel you have been placed at risk, you can contact the Chair of the Human Subjects' Institutional Review Board via email (research@fgcu.edu).

Statement: I have read the preceding information describing this study. All of my questions have been answered to my satisfaction. I am 18 years of age or older and freely consent to participate in the study. My decision to participate or to decline participating in this study is completely voluntary. I understand that I am free to withdraw from the study at any time. I am aware of my option to not answer to any questions I choose.

I understand that it is not possible to identify all potential risks I believe that reasonable steps have been taken to minimize both the known and potential but unknown risks. The submission of the completed survey is my informed consent to participate in the study.

If you would like a copy of the consent form, print a copy before continuing.

By clicking on “next” at the bottom of this page you are consenting to participate in this research survey.

If you do not wish to participate, click the “x” in the top corner of the browser to exit.

Thank you very much for your time.

Dr. Peter Reuter
Assistant Professor
Department of Rehabilitation Sciences
Marieb College of Health & Human Services

How old are you?

Race/ethnicity

Mark all that apply

- Caucasian
- African-American/black
- Hispanic
- Non-hispanic
- East Asian
- Other:

What is your relationship status?

- Single
- In a committed relationship
- In an open relationship
- Married

Are you sexually active?

- Yes
- No

How old were you the first time you were sexually active?

What is your biological sex?

- Male
- Female

What is your gender identity?

- Male
- Transgender male to female
- Transgender female to male
- Female

What is your sexual orientation?

- Hetersexual
- Homosexual (gay/lesbian)
- Bisexual
- Pansexual
- Asexual

Are you an FGCU student?

- Yes
- No

Are you a

- Freshman
- Sophomore
- Junior
- Senior
- Graduate student
- Second degree student
- Non-degree seeking student

Are you a full time student?

- Yes
- No

Are you an instate (Florida) student?

- Yes
- No

Which country (other than US) or state are you from?

Which college do you belong to?

- Arts and Sciences
- Education
- Business
- Health Professions and Human Services
- Engineering
- Other:

Do you have an emotional support or service animal?

- Yes
- No

What animal?

What is your current overall GPA?

What was your overall GPA at the end of your junior year approximately?

What was your overall GPA at the end of your sophomore year approximately?

What was your overall GPA at the end of your freshman year approximately?

What is the highest educational level attained by your father?

Please note that this question asks about the highest grade level completed, not the highest grade level attended. If a parent has attended college but not obtained a Bachelor's degree, the "High school" option should be marked.

- Did not complete high school
- High school
- Associate degree
- Bachelor's degree
- Master's degree
- Doctoral degree

What is the highest educational level attained by your mother?

Please note that this question asks about the highest grade level completed, not the highest grade level attended. If a parent has attended college but not obtained a Bachelor's degree, the "High school" option should be marked.

- Did not complete high school
- High school
- Associate degree
- Bachelor's degree
- Master's degree
- Doctoral degree

What are your living arrangements?

- ◯ On campus
- ◯ At home with parents
- ◯ Off campus with other students/roomates
- ◯ Off campus on your own

Are you a part of Greek Life at FGCU?

- ◯ Yes
- ◯ No

Are you a member of a registered FGCU student organization or club?

- ◯ Yes
- ◯ No

Are you religious?

- ◯ Yes
- ◯ No

During the past 7 days, how many hours of sleep did you get on average per night?

- ◯ 4 hours or less
- ◯ 5 hours
- ◯ 6 hours
- ◯ 7 hours
- ◯ 8 hours
- ◯ 9 hours
- ◯ 10 or more hours

During the past 7 days, on average what time did you wake up each day?

- ◯ Before 6am
- ◯ Between 6am and 8am
- ◯ Between 8am and 10am
- ◯ Between 10am and 12pm
- ◯ After 12pm

During the past 7 days, on average what time did you go to bed each night?

- ◯ Before 8pm
- ◯ Between 8pm and 10pm
- ◯ Between 10pm and 12am
- ◯ Between 12am and 2am
- ◯ After 2am

Do you have a medical condition that affects the way you sleep?

- ◯ Yes
- ◯ No

Which condition(s) affects the way you sleep?

- ☐ Anxiety Disorders
- ☐ Sleep Apnea
- ☐ Insomnia
- ☐ PTSD
- ☐ Chronic Pain
- ☐ Other:

Do you work?

- ◯ Yes
- ◯ No

On average, how many hours do you work per week?

- Under 5 hours
- 5-10 hours
- 10-20 hours
- 20-30 hours
- 30-40 hours
- Over 40 hours

On average, during the past 7 days, how many hours did you watch or read on a TV/Computer/Tablet (iBooks, Kindle, online newspaper or article, Netflix, Hulu, Amazon Prime Video, or another streaming source) per day?

- Not at all
- Less than 1 hour per day
- 1 hour per day
- 2 hours per day
- 3 hours per day
- 4 hours per day
- 5 hours or more per day

Do you check your phone at night after you are in bed?

- Yes
- No

During the past 7 days, how many hours did you play video or computer games or use a computer/tablet/smart phone for something that is not school work on average per day?

- Not at all
- Less than 1 hour per day
- 1 hour per day
- 2 hours per day
- 3 hours per day
- 4 hours per day
- 5 or more hours per day

How long before falling asleep do you turn off TV/technology?

- More than 1 hour before
- 1 hour before
- 30 minutes before
- 20 minutes before
- 10 minutes before
- 5 minutes before
- 1 minute or less before

Do you have serious difficulty concentrating, remembering, or making decisions because of a physical, mental, or emotional problem?

- Yes
- No

Have you been diagnosed with/are you being treated for a mental health disordery?

- Yes
- No

Have you been diagnosed with/are you being treated for a learning disability?

- Yes
- No

Which disorder/disability are you being treated for?

During the past 12 months, have you ever been bullied on campus including the dorms?

Bullying is when 1 or more students tease, threaten, spread rumors about, hit, shove, or hurt another student over and over again. It is not bullying when 2 students of about the same strength or power argue or fight or tease each other in a friendly way.

- Yes
- No

During the past 12 months, have you ever been cyber bullied?

Count being bullied through texting, Instagram, Facebook, or other social media.

- Yes
- No

During the past 12 months, did you ever feel so sad or hopeless almost every day for two weeks or more in a row that you stopped doing some usual activities?

- Yes
- No

The following question(s) ask about suicidal thoughts and suicide attempts. You are under no obligation to answer any of the questions but can skip over this part and continue the survey on the next page.

During the past 12 months, did you ever seriously consider attempting suicide?

- Yes
- No

How many times did you consider attempting suicide?

- 1 time
- 2 or 3 times
- 4 or 5 times
- 6 or more times

Did you make a plan about how you would attempt suicide?

- Yes
- No

Did you actually attempt suicide?

- Yes
- No

How many times did you actually attempt suicide?

- 0 times (not at all)
- 1 time
- 2 or 3 times
- 4 or 5 times
- 6 or more times

Did the attempt result in an injury, poisoning, or overdose that had to be treated by a doctor or nurse?

- Yes
- No

We understand that some participants may find it hard to answer certain questions or may get overwhelmed by emotions when describing events from the past.

If you are in immediate distress or crisis and need to speak to a clinician call the CAPS office and ask for an appointment or to speak with a clinician. Nights, weekends, and holidays, you may call the CAPS help line (239) 745-3277 and you will be connected to a clinician.

If you are imminent danger to harm yourself or others, call 911 if you are off campus and 590-1911 if you are on campus, or you may go to the emergency room at the nearest hospital.

You may also contact the National Suicide Prevention Lifeline at 1-800-273-TALK (8255).

How tall are you?

In height and inches

How much do you weigh?

In pounds

How do you describe your weight?

- Very underweight
- Slightly underweight
- About the right weight
- Slightly overweight
- Very overweight

Which of the following are you trying to do about your weight?

- I am not trying to do anything about my weight
- Lose weight
- Gain weight
- Stay the same weight

During the past 7 days, how many times did you eat vegetables?

- 0 times (not at all)
- 1 to 3 times
- 4 to 6 times
- 7 to 10 times
- 11 times or more

During the past 7 days, on how many days were you physically active for a total of at least 60 minutes per day?

- 0 days (not at all)
- 1 day
- 2 days
- 3 days
- 4 days
- 5 days
- 6 days
- 7 days

During the past 7 days, on how many days did you do exercises to strengthen or tone your muscles, such as push-ups, sit-ups, or weight lifting?

- 0 days (not at all)
- 1 day
- 2 days
- 3 days
- 4 days
- 5 days
- 6 days
- 7 days

Do you have any dietary limitations/restrictions?

- Yes
- No

Which dietary limitations/restrictions do you have?

Are you a

- [ ] Vegan
- [ ] Vegetarian
- [ ] Gluten-free
- [ ] Lactose intolerant

- [ ] Diabetic
- [ ] Other:

During the past 7 days, how many times did you drink 100% fruit juices such as orange juice, apple juice, or grape juice?

Do not count punch, Kool-Aid, sports drinks, or other fruit-flavored drinks.

- ○ 0 times (not at all)
- ○ 1 to 3 times
- ○ 4 to 6 times
- ○ 7 to 10 times
- ○ 11 times or more

How many times did you eat fruit during the last 7 days?

Do not count fruit juice.

- ○ 0 times (not at all)
- ○ 1 to 3 times
- ○ 4 to 6 times
- ○ 7 to 10 times
- ○ 11 times or more

During the past 7 days, how many times did you eat green salad?

- ○ 0 times (not at all)
- ○ 1 to 3 times
- ○ 4 to 6 times
- ○ 7 to 10 times
- ○ 11 times or more

During the past 7 days, how many times did you drink a can, bottle, or glass of soda or pop, such as Coke, Pepsi, or Sprite?

Do not count diet soda or diet pop.

- ○ 0 times (not at all)
- ○ 1 to 3 times
- ○ 4 to 6 times
- ○ 7 to 10 times
- ○ 11 times or more

During the past 7 days, how many times did you drink a can, bottle, or glass of diet soda or pop, such as Diet Coke, Diet Pepsi, or Diet Sprite?

- ○ 0 times (not at all)
- ○ 1 to 3 times
- ○ 4 to 6 times
- ○ 7 to 10 times
- ○ 11 times or more

During the past 7 days, how many times did you drink an energy drink, such as 5 Hour Energy, Red Bull, Monster, AMP, Full Throttle, etc.

- ○ 0 times (not at all)
- ○ 1 to 3 times
- ○ 4 to 6 times
- ○ 7 to 10 times
- ○ 11 times or more

During the past 7 days, how many times did you drink a can, bottle, or glass of a sports drink such as Gatorade or Powerade?

Do not count low-calorie sports drinks such as Propel or G2.

- ○ 0 times (not at all)
- ○ 1 to 3 times
- ○ 4 to 6 times
- ○ 7 to 10 times
- ○ 11 times or more

Over the past 7 days, how many cups of caffeinated coffee have you consumed on average per day?

Include hot and iced coffee

1 cup = 8oz-10oz

- 0 (none at all)
- 1-2 cups
- 3-4 cups
- 5-6 cups
- more than 7 cups

Over the past 7 days, how many cups of caffeinated tea do you drink on average per day?

Include hot and iced tea

1 cup = 8oz-10oz

- 0 cups (not at all)
- 1-2 cups
- 3-4 cups
- 5-6 cups
- 7 or more cups

How many times did you drink a bottle or glass of plain water per day?

Count tap, bottled, and unflavored sparkling water.

1 glass of water = 8oz-10oz

- less than one glass
- 1-2 glasses
- 3-4 glasses
- 5-6 glasses
- 7-8 glasses
- 8-10 glasses
- 11-12 glasses
- 13 or more glasses

During the past 7 days, how many glasses of milk did you drink?

Count the milk you drank in a glass or cup, from a carton, or with cereal.

- 0 glasses (not at all)
- 1 to 3 glasses
- 4 to 6 glasses
- 7 to 10 glasses
- 11 glasses or more

During the past 7 days, on how many days did you eat breakfast?

- 0 days (not at all)
- 1 day
- 2 days
- 3 days
- 4 days
- 5 days
- 6 days
- 7 days (everyday)

During the past 7 days, how often did you eat on campus?

- 0 times (not at all)
- 1-3 times
- 4-6 times
- 7 -10 times
- 11 times or more

During the past 7 days, how many times did you eat fast food?

- 0 times (not at all)
- 1 to 3 times
- 4 to 6 times
- 7 to 10 times
- 11 times or more

On average, how many nights a week do you go out?

- 0 (Not at all)
- 1 night
- 2 nights
- 7 nights
- 4 nights
- 5 nights
- 6 nights
- 7 nights (every night)

Have you ever tried cigarette smoking?

- Yes
- No

How old were you when you first tried cigarette smoking?

Do you consider yourself having a smoking habit?

- Yes
- No

During the past 7 days, how many packs of cigarettes did you smoke overall?

- less than 1 pack
- 1 pack
- 2 packs
- 3 packs
- 4 packs
- 5 packs
- 6 packs
- 7 packs
- Other:

Do you smoke cigars?

- Yes
- No

During the past 7 days, how many cigars did you smoke overall?

- 1-3
- 4-7
- 8-11
- 12-15
- Other:

During the past 7 days, on how many days did you use chewing tobacco, snuff, dip, snus, or dissolvable tobacco products?

For example, Redman, Levi Garrett, Beechnut, Skoal, Skoal Bandits, Copenhagen, Camel Snus, Marlboro Snus, General Snus, Ariva, Stonewall, or Camel Orbs

- 0 days (not at all)
- 1 day
- 2 days
- 3 days
- 4 days
- 5 days
- 6 days
- 7 days (everyday)

Have you ever used an electronic vapor product?

Electronic vapor products include e-cigarettes, e-cigars, e-pipes, vape pipes, vaping pens, e-hookahs, and hookah pens.

- Yes

- No

During the past 7 days, on how many days did you use an electronic vapor product?

- 0 days (not at all)
- 1 day
- 2 days
- 3 days
- 4 days
- 5 days
- 6 days
- 7 days (everyday)

What level of nicotine (mg) does your eliquid contain ?

During the past 12 months, did you ever try to quit using all tobacco products, including cigarettes, cigars, smokeless tobacco, shisha or hookah tobacco, and electronic vapor products?

- Yes
- No

Have you ever consumed alcoholic drinks?

Drinking alcohol does not include drinking a few sips of wine for religious purposes.

- Yes
- No

How old were you when you first consumed alcohol?

During the past 30 days, on how many days did you have at least one drink of alcohol?

- 0 days (not at all)
- 1 or 2 days
- 3 to 5 days
- 6 to 9 days
- 10 to 19 days
- 20 to 29 days
- All 30 days (everyday)

During the past 30 days, on how many days did you have 4 or more drinks of alcohol in a row?

- 0 days (never)
- 1 day
- 2 days
- 3 to 5 days
- 6 to 9 days
- 10 to 19 days
- 20 or more days
- All 30 days (everyday)

During the past 30 days, on how many days did you have 5 or more drinks of alcohol in a row?

- 0 days (never)
- 1 day
- 2 days
- 3 to 5 days
- 6 to 9 days
- 10 to 19 days
- 20 or more days
- All 30 days (everyday)

During the past 30 days, what is the largest number of alcoholic drinks you had in a row?

- 0 drinks

- 1 or 2 drinks
- 3 drinks
- 4 drinks
- 5 drinks
- 6 or 7 drinks
- 8 or 9 drinks
- 10 or more drinks

Have you ever used marijuana?

Marijuana also is called grass, pot, or weed.

- Yes
- No

Do you use marijuana

- [ ] recreationally
- [ ] medicinally

How old were you when you tried marijuana for the first time?

When was the last time you used marijuana?

During the past 7 days, how many times did you use marijuana?

- 0 times (not at all)
- 1 day
- 2 days
- 3 days
- 4 days
- 5 days
- 6 days
- 7 days (everyday)

How many times have you taken prescription pain medicine without a doctor's prescription or differently than how a doctor told you to use it?

Count drugs such as codeine, Vicodin, OxyContin, Hydrocodone, and Percocet.

- 0 times (not at all)
- 1 or 2 times
- 3 to 9 times
- 10 to 19 times
- 20 to 39 times
- 40 or more times

How old were you when you used prescription pain killers for the first time?

When was the last time you used prescription pain killers?

How many times have you taken steroid pills or shots without a doctor's prescription?

- 0 times (not at all)
- 1 or 2 times
- 3 to 9 times
- 10 to 19 times
- 20 to 39 times
- 40 or more times

How old were you when you used steroids for the first time?

When was the last time you used steroids?

Have you ever used drugs other than marijuana, such as cocaine, heroin, methamphetamines, ecstasy, hallucinogenic drugs, synthetic marijuana or sniffed glue, breathed the contents of aerosol spray cans or inhaled any paints or sprays to get high?

- Yes
- No

Which drug(s) have you used?

How old were you when you used any of these drugs for the first time?

When was the last time you used any of these drugs?

Thank you very much for the taking the time to complete this survey.

Let us reassure you one more time that because you submitted your answers anonymously, neither we nor anybody else who will look at individual surveys or the compiled results of all surveys will be able to identify you or any other participant in this study.

If you have any questions about this study, you may contact the Principal Investigator Dr. Peter Reuter at 239-590-7512 or via email at preuter@fgcu.edu

If you have any questions about your rights as a participant in this research, or if you feel you have been placed at risk, you can contact the Chair of the Human Subjects' Institutional Review Board through Sandra Terranova, Office of Research and Sponsored Programs, at 239-590-7522.
